# Single-molecule localization microscopy error is sensor dependent and larger than theory predicts

**DOI:** 10.1016/j.bpr.2025.100223

**Published:** 2025-07-24

**Authors:** Alfonso Brenlla, Laila Deen, Paolo Annibale

**Affiliations:** 1 School of Physics and Astronomy, University of St Andrews, St Andrews, United Kingdom

## Abstract

Since the advent of stochastic localization microscopy approaches in 2006, the number of studies employing this strategy to investigate the subdiffraction limit features of fluorescently labeled structures in biology, biophysics and solid state samples has increased exponentially. Underpinning all these approaches is the notion that the position of single molecules can be determined to high precision, provided enough photons are collected. The determination of exactly how precisely, has been demanded to formulas that try to approximate the so-called Cramer-Rao lower bound based on input parameters such as the number of photons collected from the molecules, or the size of the camera pixel. These estimates should, however, be matched to the experimental localization precision, which can be easily determined if, instead of looking at single beads, we study the distance between a pair. We revisit here a few key works, observing how these theoretical determinations tend to routinely underestimate the experimental localization precision of the order of a factor 2. A software-independent metric to determine, based on each individual setup, the appropriate value to set on the localization error of individual emitters is provided.

## Why it matters

This piece revisits a key question in single-molecule localization microscopy: how accurately do theoretical formulas estimate actual localization precision? Localization precision is expected to improve with increasing photon count and is often calculated using estimators based on photon number, pixel size, and background noise of the camera used. However, these formulas may not reflect real-world error. By repeatedly measuring distances between bright emitters, we compare empirical variance to a widely used formula. Surprisingly, using EM-CCD sensors, actual error can be found over twice the predicted value; this is largely dependent on the camera sensor, highlighting the variability across microscopes and the limitations of a universal localization precision formula.

## Introduction

The idea that a single-molecule emitter can be localized with an accuracy much higher than the diffraction limit is now a widespread notion and the cornerstone of fluorescence localization microscopy methods. This has spawned a large number of studies investigating cellular structures at high resolution (1) and it is now proposed that these approaches can begin to complement ultrastructural approaches, e.g., electron microscopy, for structural biology (2,3,4).

Formulas that calculate the localization accuracy of single emitters imaged under a microscope and onto a camera sensor are based on the optical setup parameters and the number of photons collected. An original formulation was first proposed in 2002 by Thompson et al. (5), which developed an earlier theory by Bobroff (6). Thompson et al.’s work proposed a formula to estimate the localization precision of a point emitter, and an associated computational algorithm for the fitting, based on the use of Gaussian masks. The Thompson et al. formula contains effectively three terms: one to account for photon noise, one to correct for the finite size of the pixel, and the third term to take into account background noise for the case of weak emitters. The average of the square of the localization accuracy in the photon noise-regime scales as 1/*N* with respect to the total number of collected photons, whereas in the background noise regime it scales as 1/*N*^2^. This resulted in the well-known equation:

$$<{\left(\Delta x\right)}^{2}>=\frac{\left(s^{2}+\frac{a^{2}}{12}\right)}{N}+\frac{8\pi {s^{4}b}^{2}}{a^{2}N^{2}}$$

where *a* defines the pixel size at the sample, *b* the background photons measured in the fitting window of the given emitter, and *s* is the standard deviation of the 2D point-spread function (typically a Gaussian). This formula was incorporated in the original manuscripts that in 2006 proposed PALM (7), fPALM (8), and STORM (9). Simply put, the formula shows clearly that the more photons are collected, the lower the localization error. It was used widely throughout the first years of localization microscopy research; nonetheless, a few years later, Mortensen et al. (10) suggested to revisit the formula, implying that Thompson et al.’s formula overestimated the localization error. The Mortensen et al. formula is shown below:

$$<{\left(\Delta x\right)}^{2}>=\frac{s_{}^{2}}{N}{\left(1+\int_{0}^{1}\frac{\ln\;t}{1+t/\tau }dt\right)}^{-1}\text{,}$$

with $\tau =2\pi {\sigma_{a}^{2}b}^{2}/Na^{2}$.

**Figure 1:**
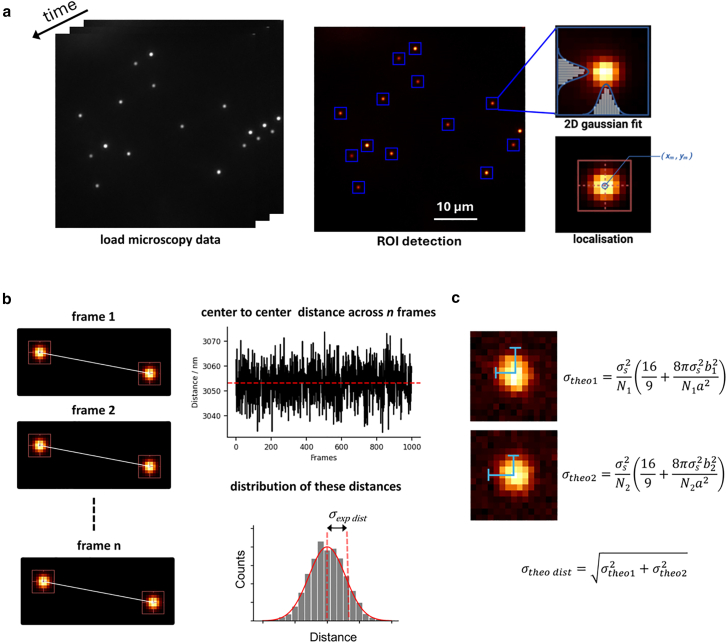
Experimental localization error from pairwise distances of single bright fluorescent emitters. (*a*) Regions of interest (ROIs) with signal above a certain threshold are identified and fitted with a 2D Gaussian PSF to yield centroid coordinates x_m_ and y_m_. (*b*) The fit parameters from (*a*) also include the number of photons in the image, which are used to calculate the theoretical localization error for each bead, which is then propagated to the error on the distance. (*c*) The subpixel centroid coordinates x_m_ and y_m_ enable the determination of interbead distances over all frames, which are then used to build a histogram of the interbead distances, which displays, as expected, a Gaussian profile. The standard deviation of the Gaussian is defined as the experimental localization error.

The Mortensen et al. approach introduced a more precise localization scheme and evaluated Thompson et al.’s error estimate as 1.78 times larger when doing least squares fitting of single emitters with Gaussian masks (Fig. 1) over maximum likelihood estimation fits using Gaussians (MLEwG), suggesting that MLEwG localization would allow getting closer to the theoretical lower localization error, known as the Cramer-Rao lower bound (CRLB) (11). Based on this limit, in the presence of an adequate number of photons, subnanometer localizations can be achieved on bright, isolated emitters, in principle allowing to study molecular interactions on spatial scales previously accessible only to single-molecule FRET.

In practice, however, colocalizations in the nm range have proved elusive, owing to the intrinsic nanoscale fluctuations of any microscopy setup as well as the pixelated nature of the detector and the noise characteristic of the camera electronics (12). In this respect, it is important to note that Mortensen et al., who measured the fluctuations in the distance between pairs of 40 nm immobilized beads (10), observed that the variance of the measured (experimental) distances appeared to be always twice the one expected from the CRLB, which was attributed to the effect electron multiplication gain of the EMCCD camera (the authors used an Andor iXon DV887 back-illuminated camera, relying on a e2V CCD97 chip). At the same time, forward scattering interferometry measurements of immobilized beads on glass surfaces allowed measuring their distance with a precision better than 0.5 nm, indicating that no fundamental problem exists in achieving the beads and setup stability necessary for such a precision (13).

This raises the question as to whether the localization precision estimate broadly used in the field, and which is used to render most single-molecule-based superresolution microscopy images, is an accurate estimator of the actual real-life localization precision. We shall note here that methods have been proposed to generate an estimate of the localization precision in superresolution data sets from the data sets themselves (14,15), albeit these approaches would incorporate any variability from sample preparation (e.g., cell chemical fixation), and therefore do not necessarily convey the fundamental resolution achievable with a given setup.

## Materials and methods

### Sample preparation

A stock solution of TetraSpeck Microspheres (catalog no. T7279, ThermoFisher Scientific, Carlsbad) with 0.1 *μ*m diameter size and concentration of 2 × 10^15^ particles per mL was diluted 100 times in a 10 mM MgCl_2_ solution and sonicated for 15 min to break particle aggregates. The bead solution was placed on a clean coverslip and incubated for 20 min to allow particles to sediment and attach onto the glass surface. After immobilization, the solution was gently pipetted out and replaced with pure water to prevent floating beads to interfere with subsequent microscopy measurements.

### Optical setup

A red laser beam (Cobolt, Solna, CW 638 nm) was expanded and passed through the back port of an Olympus (Tokyo) IX73 inverted microscope. A dichroic mirror DMLP650R (Thorlabs, Newton) redirected the laser beam toward the sample through an Olympus 60x Apo N oil objective in either wide-field epi or TIRF illumination mode (Fig. S1). The collected fluorescence emission was magnified and focused onto a camera sensor chip. The three camera models tested in this study are an Andor (Belfast) sCMOS model SONA-4BV6X, an Andor iXon Ultra DU-897U-CS0-#BV EMCCD, and a Photometrics (now Teledyne Photometrics, Birmingham) Cascade serial no. B07M892005 EMCCD. The pixel size for each camera was calculated by imaging a standard 10 *μ*m groove reticule (Muhwa Scientific, Shanghai). All optical elements were attached to a floating optical table situated in the lowest floor of the building.

### Data collection and analysis

After bead immobilization, all movies were recorded with an acquisition time of 100 ms for a length of 1000 frames. For Andor cameras, counts were converted to photoelectrons according to the manufacturer procedure. We used the sensitivity value reported in the System Performance Sheet which accompanied each respective camera, while the sensor background or bias was measured in total dark conditions. The sensitivity, bias, and gain response of the Cascade camera were determined in-house following the manufacturer’s indications. For bead localization analysis, we translated Mortensen et al.’s code directly into Python and ran it on our data sets. This method employs a maximum likelihood estimation (MLE) algorithm for fitting a 2D Gaussian to the experimental PSF. Since MLE methods are slow and very sensitive to initial parameters, we additionally ran a faster least squares regression analysis and then fed the optimized parameters onto the MLE pipeline. Once the PSF centroids positions are estimated, we determined the distance over time between all possible bead pairs. The experimental localization error is the standard deviation of the interbead pairs distance. The theoretical localization error for interbead distance was calculated through error propagation from individual bead localization errors computed from Mortensen et al.’s formula and averaged over all frames. The code pipeline employed in this analysis, together with detailed instructions on running the software is available at "GitHub: https://github.com/brenlla1/Localisation-precision".

## Results and discussion

To evaluate the accuracy of the localization error formulas commonly used in the literature, we used an EMCCD camera having a comparable sensor (Photometrics Cascade II 512) to the one used in the original manuscript of Mortensen et al. and acquired images of subdiffraction limited diameter (100 nm) fluorescent beads (Fig. 1 *a*). We reasoned that a direct measurement of the bead localization precision can be obtained from the standard deviation of the repeated measured distance between any two beads. Based on the notion, due to error propagation, that the standard deviation of the measured distance between any pair of beads arises from the sum in quadrature of the localization precision of each individual bead ($\sigma_{theo}=\sqrt{\sigma_{theo\;1}^{2}+\sigma_{theo\;2}^{2}}$), we compared the experimental localization precision of a pair of beads (σ_exp_) (Fig. 1 *b*) to the sum in quadrature of the bead localization precision calculated according to Mortensen et al. (σ_theo_) (Fig. 1 *c*). Ideally, if the localization precision formula reported in the literature is accurate, the two values should match.

The basic idea behind the distance measurement is to conduct a repeated measurement of a quantity whose measurement error (standard deviation) is dependent on the localization precision of the beads, namely the center to center distance between the two beads. Any repeated measurement of the same physical quantity (measurement of a rod, of the duration of the oscillation of a pendulum, etc.) will yield a Gaussian distribution of values around a mean value, the mean reflecting the so-called “true” quantity and the standard deviation corresponding to the experimental error. If a variable depends on measuring two observables, then the error on said variable can be calculated through error propagation (16). The use of distance between two beads, in the specific case of our work, has additionally the advantage of making the measurements largely drift independent (Fig. S2), since all the beads in a field of view present a similar drift pattern, which does not alter interbead distances.

**Figure 2:**
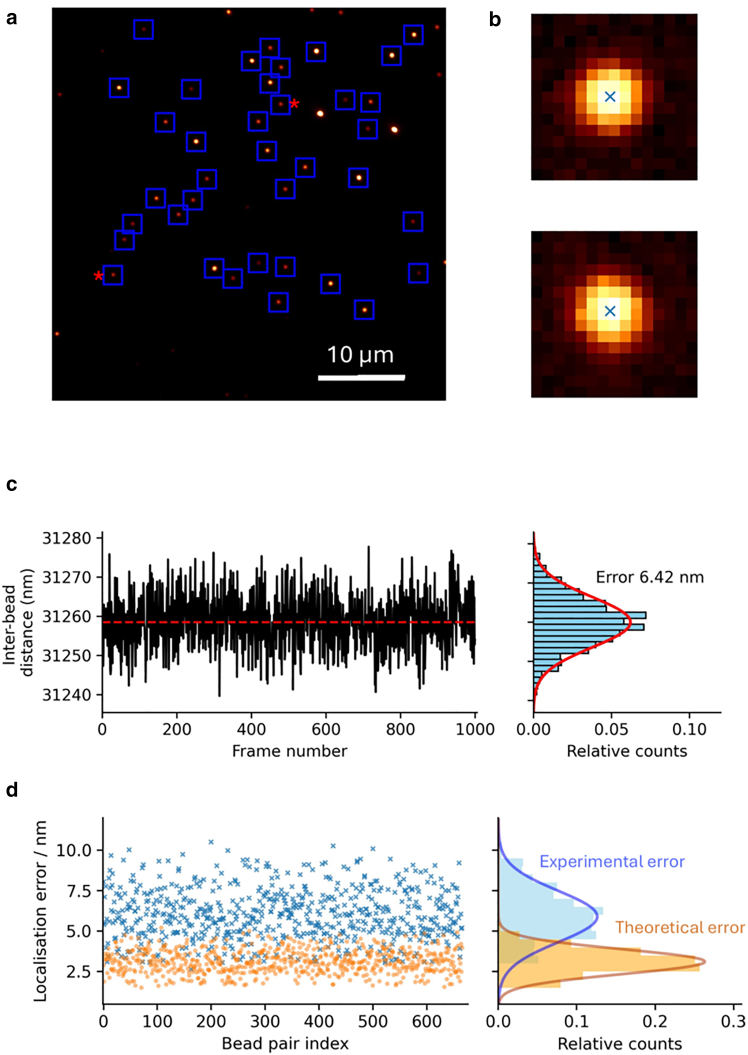
Experimental and theoretical errors for an EMCCD Cascade camera with gain disabled. (*a*) Field of view with regions of interest containing individual beads marked as blue squares. (*b*) Centroid positions (*blue cross*) obtained via MLE fitting for the beads marked with a red asterisk (∗) in (*a*). (*c*). Distance over time for the two beads shown in (*b*) and the corresponding histogram with a Gaussian fit shown in red. (*d*). Experimental and theoretical localization errors for all the bead pairs measured using an EMCCD Cascade with gain disabled and their corresponding histograms.

To fit the beads position (x_m_, y_m_) we used the MLEwG algorithm and code reported by the authors (10) (Fig. 2, *a* and *b*), as well as a least squares approach, which allowed us to measure bead-to-bead distances over movies containing 1000 frames. The Gaussian fits to the data proved to be—at least qualitatively—satisfactory, as shown by the lack of obvious features in the residual plots (Fig. S3). The interbead distance (Fig. 2 *c*) oscillates around a central value over time and follows a Gaussian distribution whose standard deviation corresponds to the experimental localization error (σ_exp_) for that given interbead distance. Both experimental and theoretical localization errors over all bead pairs follow Gaussian distributions (Fig. 2 *d*), with both variance and mean values larger for the experimental set.

**Figure 3:**
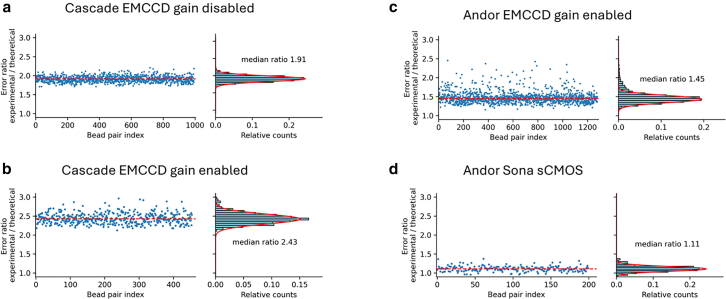
Ratio between experimental and theoretical localization errors for different optical and detection setups. (*a*) Data for a Photometrics EMCCD camera with the gain disabled, the error ration is obtained dividing the data points in Fig. 2*d*. (*b*–*d*) Depict the error ratios for a Cascade with the gain enabled, an Andor EMCCD, and an Andor Sona sCMOS.

The ratios between experimental (σ_exp_) and theoretical localization (σ_theo_) for the bead pairs are therefore not the same, and it appears that σ_theo_ underestimates significantly the actual localization error of the individual beads, albeit the relationship between the two is linear (Fig. S4). To understand better the source of this discrepancy, we explored how this metric changed when using different settings or sensors. The first observation is that the errors ratio varies depending on the sensor used (Fig. 3). The Cascade camera has two readout amplifiers that can be switched by software, one with electron charge multiplication gain (emGain) and another for traditional readout without emGain. The presence of these two readout modes within the same sensor allows for a direct measurement of any effect of emGain on the data noise and thus the localization error. With emGain enabled, the ratio between experimental and theoretical error for the Cascade camera is ∼2.4 (Fig. 3 *b*), while disabling emGain brings the ratio down to ∼1.9 (Fig. 3 *a*). Notably, this appears to suggest that the factor of 2 in the ratio σ_exp_/σ_theo_ reported by Mortensen et al. does not arise from additional noise inherent to the electron multiplication process.

Interestingly, a more modern EMCCD camera (Andor iXon897) with comparable pixel size and emGain enabled (Table S1), presents lower σ_exp_/σ_theo_ ratio with a value of ∼1.5 (Fig. 3 *c*). Overall, these results suggest that at least part of the excess experimental error arises from the camera electronics, albeit not from the actual electron multiplication process per se.

This appears supported by the fact that the use of an sCMOS sensor, which showed the lowest σ_exp_/σ_theo_ ratios, with a value of ∼1.1 (Fig. 3 *d*). No significant changes were observed on the σ_exp_/σ_theo_ ratios when using the same sCMOS sensor with pixel sizes of 34.2, 108.3, and 216.6 nm, or different excitation wavelengths (Fig. S5), These findings indicate that σ_exp_/σ_theo_ ratios are largely sensor dependent. However, it has been suggested that a different treatment of noise and calibration of the sensor individual pixels is necessary for optimal fit of sCMOS data (17). As expected, both theoretical and experimental interbead distance errors are inversely proportional to the total number of detected photoelectrons (Fig. S6, *a* and *b*), but the σ_exp_/σ_theo_ ratio does not present any clear correlation with the number of photoelectrons (Fig. S6 *c*). The distribution of photons between the two beads for a given bead pair clearly affects the localization error (Fig. S7 *a*), with the error being minimized when the number of photons is equally distributed between the two beads. Different background noise for each bead did not have any significant impact on the localization error (Fig. S7 *b*), indicating that we are working at a high photon count regime where background noise is negligible. Additionally, we did not observe any fluorophore blinking or photobleching, as our localization error remained constant throughout the movie recordings (Fig. S8).

The range of σ_exp_/σ_theo_ ratios exhibited by different camera sensors presented in this work, as well as the observation that they are all larger than one, highlights the pitfalls associated to using formulas for the localization precision that strive to approximate the CRLB, as this appears to routinely lead to an underestimation of the effective localization error. Other approaches, which, for example, rely on the use of a PSF model calibrated on the actual setup, would allow in principle a more precise estimation of localization precision, but rely on ad hoc software (18,19), and may not consider other sources of error, such as instabilities of the setup, and in particular of the emission optics. We tested the software reported in Li et al. (18), which fits the single-molecule peaks to the experimental PSF of the system determined from a set of calibration beads, and measured a σ_exp_/σ_theo_ ratio of the order of ∼2.9 for the Cascade EMCCD with emGain enabled, suggesting that, also in this case, the CRLB estimate substantially underestimates the actual localization error of the system (Fig. S9). These results overall indicate that an accessible method to provide a software-independent estimation of the localization precision of a setup, given any arbitrary code and pipeline for molecular localization, is still somehow missing.

We argue here that a straightforward procedure is a calibration for each microscope that determines the σ_exp_/σ_theo_ ratio. To that end, we have developed a basic pipeline (Fig. S10): in brief, a set of subdiffraction limit fluorescent beads is imaged repeatedly over time (Fig. 1 *a*). Then, each bead is fit with the software of choice that the researcher desires to use in their subsequent localization microscopy experiments (Fig. 1 *b*). The standard deviation of the distance between each bead pair is then calculated (Fig. 1 *d*), and this value (σ_exp_) is compared with the expected standard deviation (σ_theo_) obtained by using error propagation from the individual bead localization precision obtained using the formula of choice (e.g., Thompson et al. or Mortensen et al.) (Fig. 1 *c*). This basic procedure is aided by an open-source analysis pipeline that relies on standard Python and does not require a GPU. Given that the experimental error is directly proportional to the theoretical one (Fig. S4), once the analysis is finalized, the computed σ_exp_/σ_theo_ ratio allows for the calculation of the real localization precision of each bead σ_theo_ obtained from the formula of choice.

A recent review by Lelek et al. (1) identified five widely used SMLM software suites, which add to the number of individual, custom-made algorithms used by dozens of laboratories. This number is likely to grow, considering the impact of deep-learning approaches. The proliferation of ad hoc software for analysis, and the increase in their complexity, may have reached a point where the determination of which to choose may become challenging for a researcher. Imaging technology is also evolving and technical performance of new sensors likely to change. Finally, each individual setup has its own fingerprint in terms of optical aberrations and mechanical stability. The use of the σ_exp_/σ_theo_ ratio that we have highlighted above as a figure of merit to determine the effective localization precision of a setup appears as a software- and setup-independent approach to assess in a straightforward way the effective localization precision of single emitters, a cornerstone of the growing galaxy of superresolution microscopy approaches.

## Acknowledgments

P.A. gratefully acknowledges support from the 10.13039/501100000275Leverhulme Trust (RL-2022-015) and UKRI BBSRC IAA.

## Author contributions

A.B.-L. and P.A. designed the research. A.B.-L. and L.D. performed the research. P.A. and A.B.-L. wrote the paper with input from L.D. P.A supervised the project and acquired funding.

## Declaration of interests

The authors declare no competing interests.

## Supporting material

Document S1. Figures S1–S10 and Table S1

Document S2. Article plus supporting material
