# Supplementary material for: Single-molecule localization microscopy error is sensor dependent and larger than theory predicts
Source: Biophys Rep (N Y). 2025 Jul 24;5(3):100223. doi: 10.1016/j.bpr.2025.100223 (PMC12347845; doi:10.1016/j.bpr.2025.100223)
Supplement: Document S1. Figures S1–S10 and Table S1 [file mmc1.pdf]

Biophysical Reports, Volume *5*

Supplemental information

Single-molecule localization microscopy error is sensor dependent and larger than theory predicts

Alfonso Brenlla, Laila Deen, and Paolo Annibale

Single molecule localization microscopy error is sensor-dependent and larger than theory predicts

Alfonso Brenlla-Lopez[1], Laila Deen[1], Paolo Annibale[1,*]
School of Physics and Astronomy, University of St Andrews, United Kingdom
*correspondence to pa53@st-andrews.ac.uk

Supplementary Tables and Figures

Camera model	λ_{exc} / nm	Pixel size / nm	Pixel area	Electron multiplication	Median experimental error	Median theoretical error	Median ratio σ_{exp} / σ_{theo}
Photometrics Cascade Serial No **B07M892005**	638	89	512 x 512	Yes	6.13	2.52	2.43
				Yes	7.29	2.47	2.95*
				No	5.95	3.12	1.91
Andor iXon Ultra **DU-897U-CS0-#BV**	638	85.4	512 x 512	Yes	5.16	3.56	1.45
Andor sCMOS **SONA-4BV6X**	638	34.2	1024 x 1024	No	4.68	4.24	1.11
		108.3		No	6.77	6.23	1.09
		216.3		No	6.48	5.98	1.09
	488	108.3		No	4.44	3.90	1.14

*Fit carried out using an experimental psf model.

Table S1. Optical and electronic acquisition parameters for all measurements shown in this work.

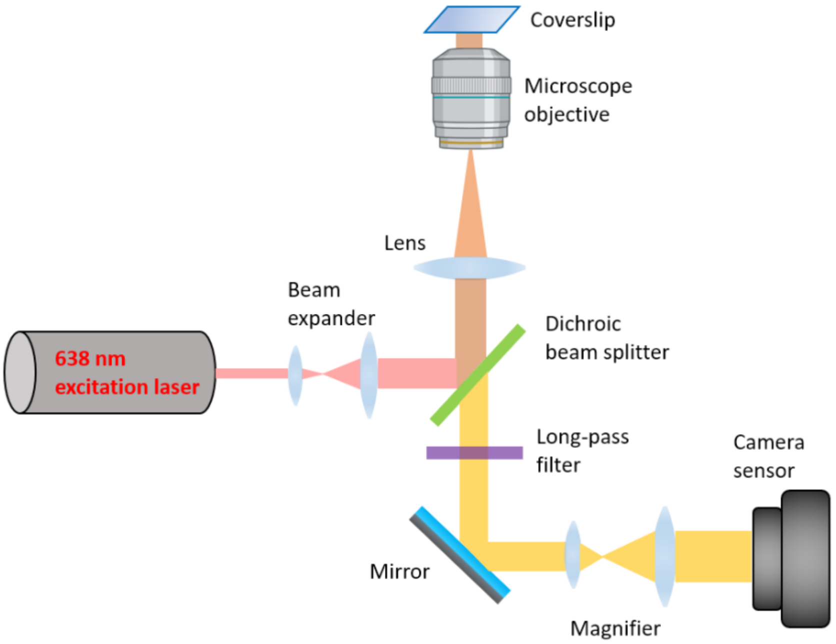

Figure S1. Schematic of the optical setup used in this work.

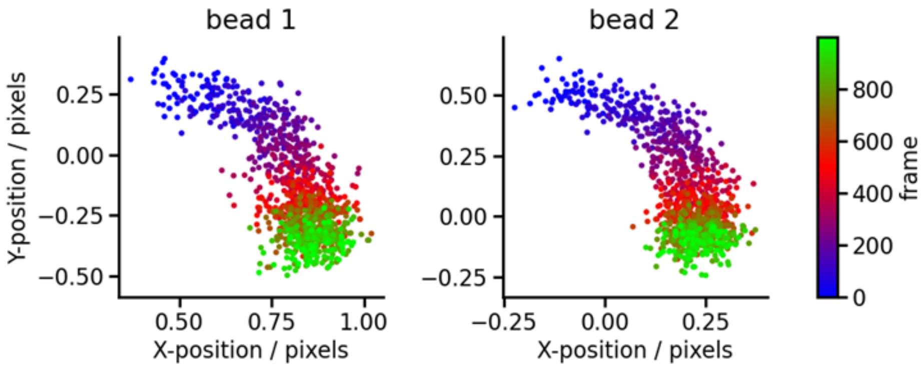

Figure S2 . Center position of a selected bead pair over time obtained from the MLE fit of Gaussian PSFs. The two beads drift about half a pixel in both x- and y- directions throughout the measurement from their starting position (blue dots) to their final position (green dots). This measurement was carried out using a Cascade EMCCE with a pixel size of 89 nm.

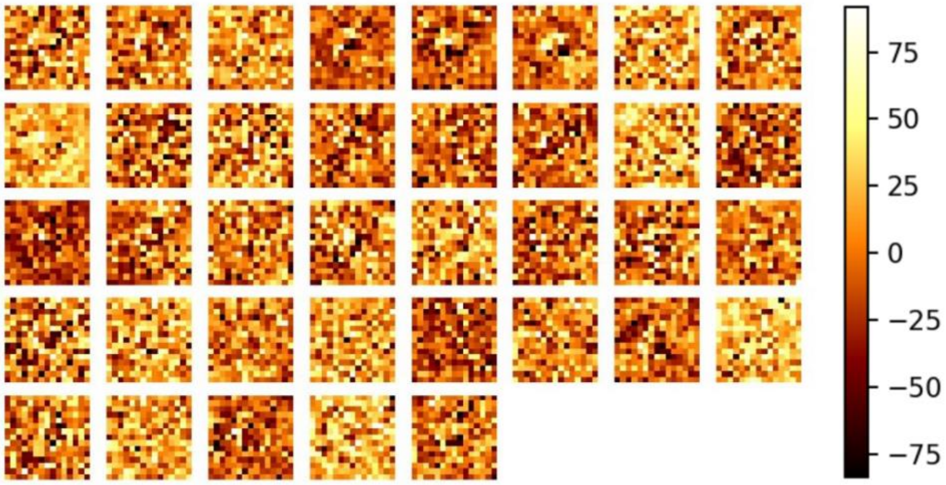

Figure S3. Representative residual plots from fits of multiple beads imaged using the Photometrics Cascade camera without emGain. Squares are 1.33 µm in side.

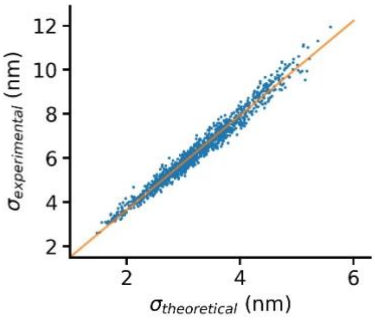

Figure S4. The experimental localisation error (y-axis) is proportional to the theoretical error (x-axis), the orange line corresponds to a linear fit. The data shown here were recorded in the Photometrics Cascade camera with EM Gain disabled.

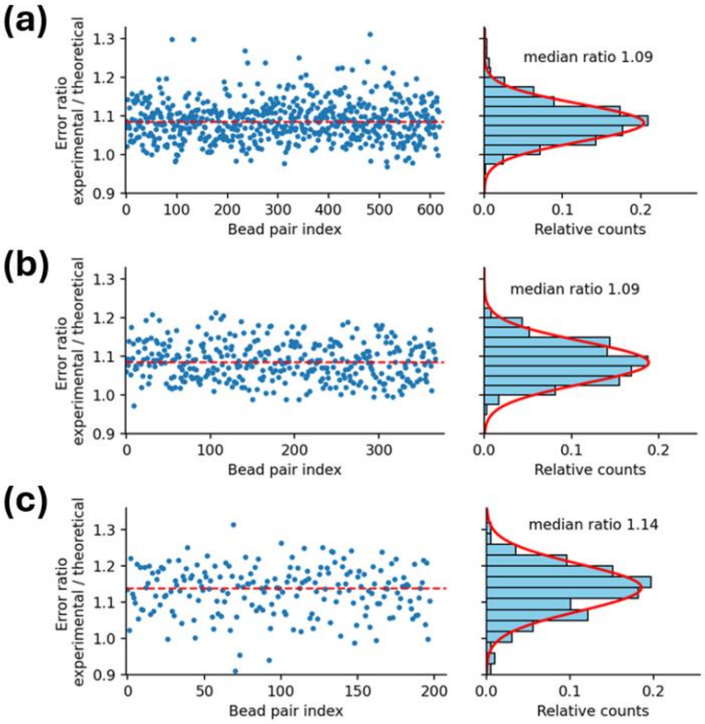

Figure S5. Ratio between experimental and theoretical localisation errors for measurements carried out on sCMOS camera with 108.3 nm **(a)** and 216.6 nm **(b)** pixel sizes. **(c)** shows the same ratio for a pixel size of 108.3 nm using a 488 nm laser for excitation. The rest of experiments in this manuscript were carried out using a 638 nm laser for excitation.

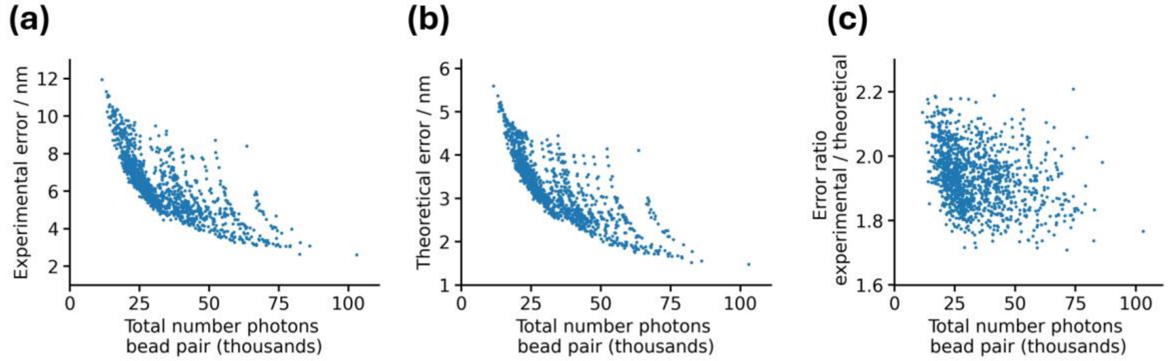

Figure S6. Experimental **(a)** and theoretical **(b)** errors for inter-bead separation as a function of the number of photons for the data recorded in the Photometrics Cascade with EM Gain disabled. **(c)** Ratio between the experimental and theoretical error shown in (a) and (b) respectively. The total number of photons corresponds to the sum of photons detected from each bead in each bead pair.

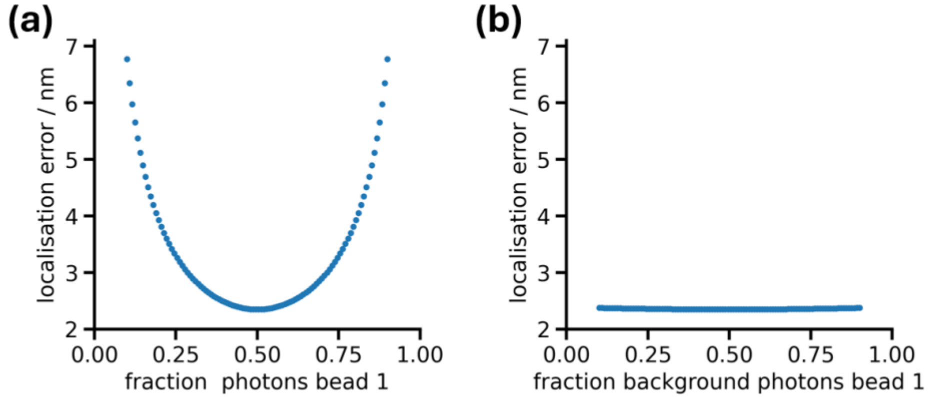

Figure S7. Effect of photon distribution and background noise on localisation error. Simulated interbead distance localisation error as a function of the number of photons in each bead **(a)** and the background noise on each bead **(b)**. For this theoretical simulation, we used the typical parameters for measurements carried out on an EMCCD Cascade with no gain, that is, 89 nm per pixel, 30,000 photons total, 100 background photons and a psf width of 1.9 pixels.

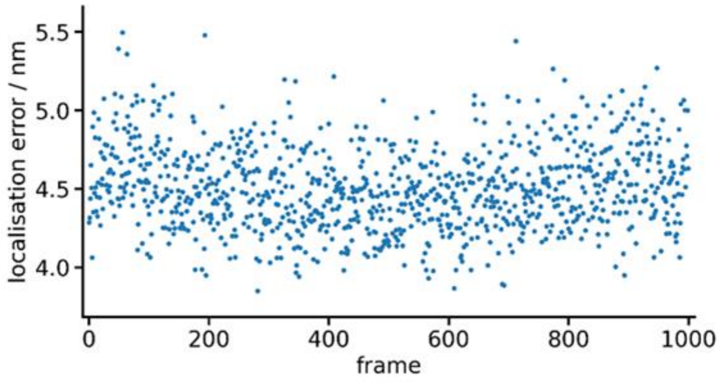

Figure S8. Theoretical localisation error (calculated using Mortensen formula) as a function of frames for a selected bead. The error is stable, indicating that with the conditions used photobleaching, leading to a reduction of N photons, and thus an increase in localisation error, is not of concern.

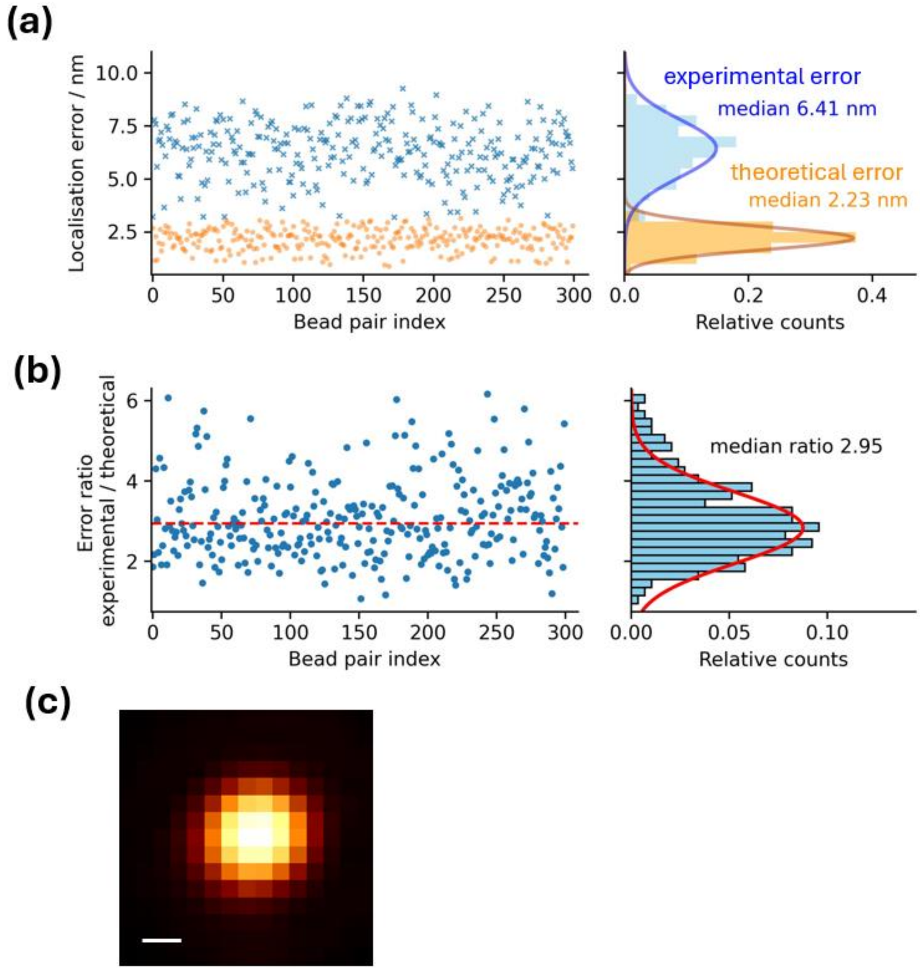

Figure S9. Experimental and theoretical localization errors using an experimental PSFmodel. **(a)** Experimental and theoretical localisation errors for over 300 bead pairs measured using the Cascade camera with an emGain value of 3,500. **(b)** Ratio between experimental and theoretical ratio for the beads shown in (a). **(c)** Image of the experimental PSF, scale bar 200 nm.

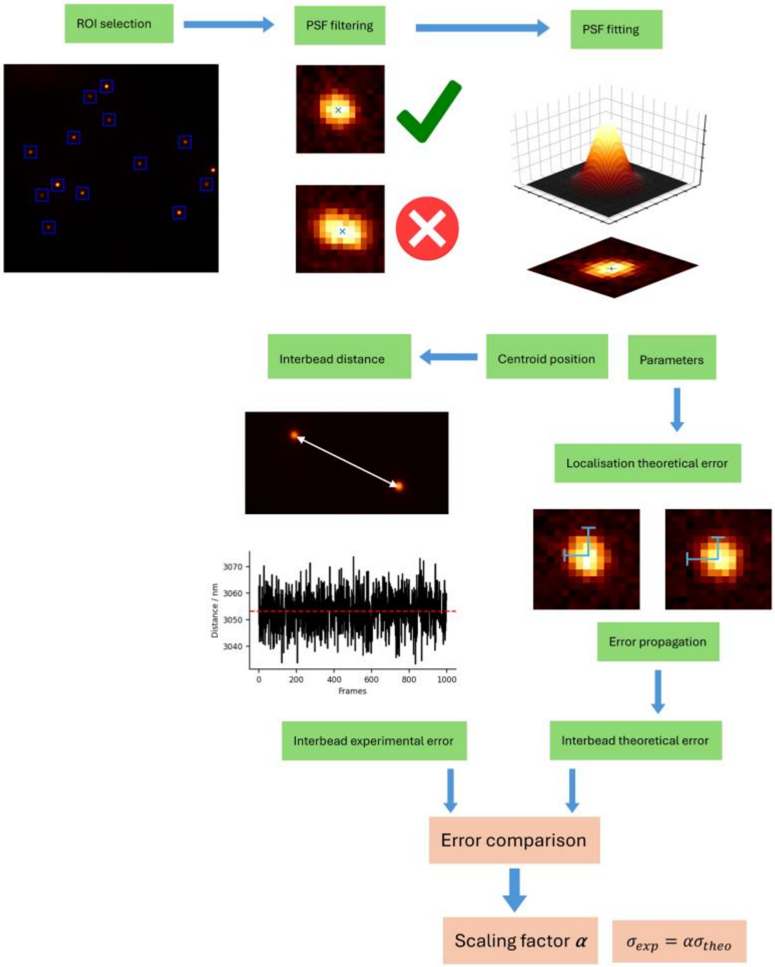

Figure S10. Data pipeline for obtaining both the experimental and theoretical errors.
